# Epitope Presentation of Dengue Viral Envelope Glycoprotein Domain III on Hepatitis B Core Protein Virus-Like Particles Produced in *Nicotiana benthamiana*

**DOI:** 10.3389/fpls.2019.00455

**Published:** 2019-04-16

**Authors:** Ee Leen Pang, Hadrien Peyret, Alex Ramirez, Hwei-San Loh, Kok-Song Lai, Chee-Mun Fang, William M. Rosenberg, George P. Lomonossoff

**Affiliations:** ^1^School of Biosciences, University of Nottingham Malaysia, Semenyih, Malaysia; ^2^Department of Biological Chemistry, John Innes Centre, Norwich, United Kingdom; ^3^iQur Limited, London, United Kingdom; ^4^Faculty of Biotechnology and Biomolecular Sciences, Universiti Putra Malaysia, Serdang, Malaysia; ^5^Division of Biomedical Sciences, School of Pharmacy, University of Nottingham Malaysia, Semenyih, Malaysia

**Keywords:** virus-like particles, epitope display, hepatitis B core antigen, dengue envelope glycoprotein, envelope glycoprotein domain III, dengue vaccine, tandem core technology

## Abstract

Dengue fever is currently ranked as the top emerging tropical disease, driven by increased global travel, urbanization, and poor hygiene conditions as well as global warming effects which facilitate the spread of *Aedes* mosquitoes beyond their current distribution. Today, more than 100 countries are affected most of which are tropical Asian and Latin American nations with limited access to medical care. Hence, the development of a dengue vaccine that is dually cost-effective and able to confer a comprehensive protection is ultimately needed. In this study, a consensus sequence of the antigenic dengue viral glycoprotein domain III (cEDIII) was used aiming to provide comprehensive coverage against all four circulating dengue viral serotypes and potential clade replacement event. Utilizing hepatitis B tandem core technology, the cEDIII sequence was inserted into the immunodominant c/e1 loop region so that it could be displayed on the spike structures of assembled particles. The tandem core particles displaying cEDIII epitopes (tHBcAg-cEDIII) were successfully produced in *Nicotiana benthamiana* via *Agrobacterium*-mediated transient expression strategy to give a protein of ∼54 kDa, detected in both soluble and insoluble fractions of plant extracts. The assembled tHBcAg-cEDIII virus-like particles (VLPs) were also visualized from transmission electron microscopy. These VLPs had diameters that range from 32 to 35 nm, presenting an apparent size increment as compared to tHBcAg control particles without cEDIII display (namely tEL). Mice immunized with tHBcAg-cEDIII VLPs showed a positive seroconversion to cEDIII antigen, thereby signifying that the assembled tHBcAg-cEDIII VLPs have successfully displayed cEDIII antigen to the immune system. If it is proven to be successful, tHBcAg-cEDIII has the potential to be developed as a cost-effective vaccine candidate that confers a simultaneous protection against all four infecting dengue viral serotypes.

## Introduction

The alarming rise of dengue epidemics has been highlighted to affect over 40% of the world population (Brady et al., 2012). The disease can be manifested as undifferentiated dengue fever or life-threatening conditions such as dengue haemorrhagic fever (DHF) and dengue shock syndrome (DSS) (Murrell et al., 2011). Classified under the Flaviviridae virus family, dengue virus (DENV) is a single-stranded, positive-sense non-segmented RNA virus with 40–50 nm enveloped particles (Guzman et al., 2010). The 10.6 kbp viral genome encodes a polypeptide that is processed into structural proteins [capsid (C); envelope glycoprotein (E); and precursor membrane (prM)] and non-structural biomolecules (NS1, 2A, 2B, 3, 4A, 4B, and 5) (Henchal and Putnak, 1990). During virus assembly, the C protein encapsidates the viral RNA to form nucleocapsid particles whereas the prM assists the folding of surface-exposed E glycoprotein (Whitehead et al., 2017). Transmission of the endemic virus has been observed in over 100 countries. The incidence rate has expanded 500-fold, spreading from South-east Asia to the Americas and Western Pacific within just a-half century (Pang and Loh, 2016). Global distribution of dengue disease is strongly influenced by urbanization, demographic, and environmental factors including global warming which has enabled *Aedes* mosquitoes to survive beyond their usual distribution. Concerns are also driven by increasing movement of travelers (Pang and Loh, 2016). The annual incidence has grown dramatically in recent decades, in which 390 million cases are predicted per annum and 96 million amongst these cases manifest an apparent clinical or sub-clinical severity (Bhatt et al., 2013). Out of these, it was reported that 500,000 people were hospitalized with severe dengue and approximately 2.5% of them would succumb to the disease (World Health Organization [WHO], 2017). The reported figure may be under-estimated due to the passive surveillance system adopted by many countries (Runge-Ranzinger et al., 2014).

To date, no specific medication is available for dengue treatment. Current clinical practices mainly rely on administration of paracetamol and intravenous fluid, together with close monitoring of the haematocrit and platelet levels (Anfasa et al., 2015). The absence of specific drugs and lack of confidence in currently marketed vaccines have also driven the public’s reliance on folk remedies that are yet to be scientifically proven. Thus, development of a dengue vaccine is still being aggressively pursued to address the unmet medical needs of people in tropical regions (Pang and Loh, 2017). For subunit vaccine production, the dengue E glycoprotein has been the most studied antigenic determinant. Its structure is organized into three ectodomains (EDIII), and serves to assist attachment and entrance into host cells via receptors (Faheem et al., 2011). The immunoglobulin-like domain III (EDIII) is an ideal immunogen as it harbors receptor binding motifs that can elicit neutralizing monoclonal antibodies production (Crill and Roehrig, 2001). In recent years, EDIII has been expressed as a consensus sequence (cEDIII) aligned between four DENV serotypes (Chiang et al., 2011; Kim et al., 2012, 2015, 2016, 2017; Huy and Kim, 2017). A proof-of-concept study showed that cEDIII could inhibit the infectivity of four dengue serotypes simultaneously following mice immunization (Leng et al., 2009). Therefore, this sequence was adopted with the aim of conferring protection against all four co-circulating dengue serotypes.

Virus-like particles (VLPs) have gradually emerged as vaccine delivery vehicles that are spontaneously assembled from viral structural proteins. These are multimeric structures that can directly stimulate immune cells by mimicking the three-dimensional conformation of native viruses. Moreover, VLPs are devoid of infectious genetic material which makes them inherently safer than attenuated or inactivated virus preparations (Pang, 2018). VLPs are known to elicit higher B- and T-cell immune responses and hence lower dosage is usually sufficient (Gamvrellis et al., 2004; Roy and Noad, 2008). The repetitive array of protein subunits in VLPs has the potential to confer superior properties as a stand-alone vaccine when compared to those of recombinant subunit-based ones which may be weak immunogens despite the use of an adjuvant (Noad and Roy, 2003). All these features make VLPs a premium platform for the production of a safe and effective vaccine (Jain et al., 2015).

In this study, hepatitis B core antigen (HBcAg) was exploited for cEDIII epitope display. Early work on HBcAg was done by Clarke et al. (1987) to produce foot and mouth disease virus fusion particles with proven serological response in guinea pigs. The icosahedral VLPs are shaped by association of two HBcAg monomers into 90 (*T* = 3) or 120 (*T* = 4) dimers, with a hairpin structure bridged by c/e1 loops to form protruding spike (Crowther et al., 1994). The flexibility of inserting foreign sequence into the immunodominant c/e1 loop for surface exposure, while retaining its antigenic properties, is ideal for antigen presentation (Pumpens and Grens, 1999). In fact, insertion in the c/e1 region was shown to impart a stronger protective response compared to N- and C-terminal fusions to the core particles (Koletzki et al., 2000). Thus, the cEDIII gene was incorporated into the c/e1 loop for the benefit of maximized exposure on protruding spikes of the assembled VLPs. “Tandem Core” technology was adopted here to produce the chimeric HBcAg VLPs. It has been shown that this technology which covalently links two core proteins into dimer forms (Peyret et al., 2015) can alleviate potential steric hindrance between two inserts at each immunodominant c/e1 loop of the dimer interface and thus promotes chimeric VLP assembly. This technology has recently been applied successfully to make other viral vaccine candidates (Ramirez et al., 2018).

In the context of molecular pharming, plants have certain advantages when compared to bacterial and animal expression systems relating to lower production cost, rapid scalability, biocontainment warranty, and eukaryotic processing machinery (Sack et al., 2015; Tschofen et al., 2016; Loh et al., 2017). The emergence of transient expression systems has ultimately sped up the process, whereby rapid candidate screening and large-scale production are achievable within days (Thuenemann et al., 2013). Moreover, there is evidence that plants may be a better choice than bacteria for the production of tandem core-based VLPs (Peyret et al., 2015). This study aimed to demonstrate the production of chimeric HBcAg particles displaying dengue cEDIII epitopes (tHBcAg-cEDIII) using a plant-based system, with the hope of developing a novel VLP-based vaccine against the deadly dengue disease. Given that DENV clade replacements had been detected in recent years (Teoh et al., 2013), the development of a vaccine that can provide a consistent protection in the long run will be highly valued (Pang, 2018). Moreover, generating a vaccine based on the single consensus cEDIII antigen should reduce the underlying cost as it obviates the need to test the best tetravalent formulation from monovalent components of each DENV serotype.

## Materials and Methods

### Recombinant Vector Construction

**FIGURE 1:**
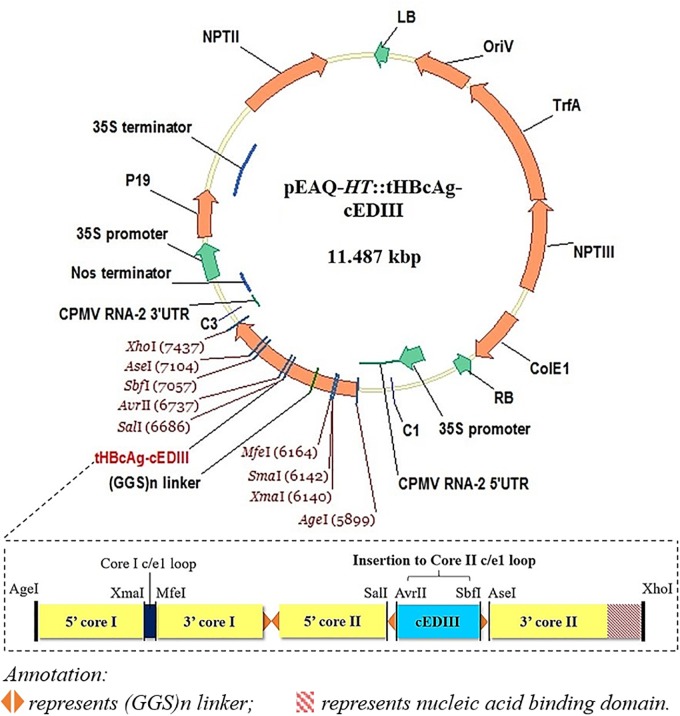
A schematic illustration of the recombinant vector, pEAQ-*HT*::tHBcAg-cEDIII. The *cEDIII* gene sequence was specifically inserted into the Core II c/el loop. The pEAQ-*HT* vector used in this study was based on Sainsbury et al. (2009), and the amino acid sequence of tHBcAg-cEDIII is shown in the Supplementary Material.

The 103 amino acid residues of cEDIII consensus sequence used (see Supplementary Material) were based on the alignment of four DENV serotypes as described by Leng et al. (2009). The synthesized gene sequence of *cEDIII* was codon-optimized (GeneArt, United States) for the expression in *Nicotiana benthamiana*. Primers were designed to incorporate *Avr*II and *Sbf*I restriction sites (underlined) at 5′ and 3′ ends of the *cEDIII* gene (cED3F: 5′-GAATACCTAGGAAGGGAATGTCATACGCTATGTGTACTGGAAAG-3′; cED3R: 5′-CATTGCCTGCAGGTGAAGATCCCTTCTTGAAC-3′) for sub-cloning into pEAQ-*HT*::tHBcAg-VHH2, a plasmid derived from pEAQ-τGFP (GenBank accession number KM396759, Peyret et al., 2015) which contains long glycine-rich linkers [(GGS)n] with unique restriction sites at the c/e1 loop region of the downstream second core (Core II) for ease of cloning. Following heat-shock transformation of competent *Escherichia coli*, putative clones harboring the expression vector (pEAQ-*HT*::tHBcAg-cEDIII) were screened and verified by sequencing (Eurofins, Germany). Figure 1 illustrates the expression cassette and corresponding recombinant vector used in this study. Additional information on the construct sequence can be found in the Supplementary Material.

### *Agrobacterium tumefaciens* Transformation and Plant Infiltration

*Nicotiana benthamiana* plants were grown on custom-mixed soil comprising of peat, 2.5 kg/m^3^ dolomite limestone, 1.3 kg/m^3^ base fertilizer, 2.7 kg/m^3^ Osmocote^®^ (applied every 3–4 months), 0.3 kg/m^3^ Exemptor^®^, and 0.25 kg/m^3^ wetter in a controlled environment of 16-h photoperiod generated by 400 W sodium lamps, 24°C and 70% relative humidity. Plants at 5–6 weeks old (until they reached the pre-flowering stage) were used in the study. The recombinant vector, pEAQ-*HT*::tHBcAg-cEDIII was introduced into *Agrobacterium tumefaciens* strain LBA4404 via electroporation. Transformed colonies were then selected from agar plates supplemented with 50 μg/ml kanamycin and 50 μg/ml rifampicin. The protocol from Sainsbury et al. (2012) was adopted here. Agrobacterial suspensions were cultured at 28°C in a shaking incubator (200 rpm) for 24–48 h. The agrobacterial cells were harvested by 4,000 × *g* centrifugation for 10 min and resuspended in MMA solution [10 mM MES (2-[N-morpholino]ethanesulfonic acid) at pH 5.6, 10 mM MgCl_2_ and 100 μM acetosyringone] to a final OD_600_ of 0.4. The abaxial side of the developed *N. benthamiana* leaves was pricked and infiltrated with the agrobacterial suspensions using a needleless syringe. Time-course evaluation of the selective leaves of infiltrated plants was performed until 9 days post-infiltration (dpi). Control agrobacterial suspensions containing the empty pEAQ-*HT* vector (Sainsbury et al., 2009) without a gene insert was included to compare with pEAQ-*HT*::tHBcAg-cEDIII for physical observation of plants post-infiltration.

### Protein Extraction

Small-scale extraction was conducted to test for protein expression and accumulation. Approximately 100 mg of the infiltrated leaf was harvested and homogenized with a ¼-inch ceramic bead (MP Biomedicals, United States) in 3× volume of extraction buffer [100 mM sodium phosphate at pH 6.8, 150 mM NaCl, 0.1% Triton-X and protease inhibitors (Roche, Switzerland)]. The Omni Bead Ruptor 24 homogeniser (Camlab, United States) was adjusted at speed setting 4 for 30 s for tissue homogenization. Samples were then centrifuged at 16,000 × *g* for 10 min and the supernatant was kept as soluble protein (SP) fraction. Nevertheless, insoluble protein (IP) fraction could be extracted from the pellet through boiling with protein denaturing buffer [NuPAGE LDS buffer (Life Technologies) mixed 3:1 with 2-mercaptoethanol] and centrifugation at 16,000 × *g* for 10 min. To check for protein integrity, sodium dodecyl sulfate polyacrylamide gel electrophoresis (SDS-PAGE) was conducted at 200 V for 50 min and stained with InstantBlue (Expedeon, United Kingdom).

For large-scale extraction, infiltrated leaves were excised, weighed and homogenized with 3× volume of the same extraction buffer in a Waring blender. Crude extracts were filtered through a layer of Miracloth before subjecting to centrifugation (20,000 × *g*) at 4°C for 20 min using an SS34 rotor (Thermo Fisher Scientific, United States). The clarified supernatant was filtered over 0.45 μm syringe filters prior to subsequent purification procedures.

### Purification of Virus-Like Particles (VLPs)

Virus-like particles samples were subjected to a two-stage purification process as described by Peyret (2015). Firstly, clarified extracts were overlain above different concentrations of sucrose solution; specifically 6 ml of 25% (w/v) and 2 ml of 70% (w/v). Double-layered sucrose cushions were prepared in UltraClear ultracentrifuge tubes (Beckman Coulter, United States) and centrifuged in a Surespin 630/36 swing-out rotor (Thermo Fisher Scientific, United States) at 167,000 × *g*, 4°C for 2.5 h. The gradient was fractionated by piercing the bottom of the tube with a needle and recovering the bottom and interface fractions. These fractions were then dialyzed thoroughly against 200 mM ammonium bicarbonate buffer (pH 8.0) overnight. Next, samples were concentrated using SpeedVac (Thermo Fisher Scientific, United States), and loaded onto a Nycodenz step gradient extending from 60 to 20% (w/v), with 2 ml of each concentration in UltraClear ultracentrifuge tubes (Beckman Coulter, United States). High-speed centrifugation was operated at 274,000 × *g* using the TH-641 swing-out rotor (Thermo Fisher Scientific, United States) for 20 h at 4°C. Bottom of the tubes was punctured with a needle and divided into successive fractions. These fractions were assayed with Western blotting to determine the distribution of the desired particles.

### Purification of cEDIII Protein

Recombinant cEDIII protein was used in this study as an antigen positive control for immunization work. In brief, the plant-expressed protein was harvested at 6 dpi following agroinfiltration with pEAQ-*HT*::PR1a-cEDIII-sGFPH-KDEL (Pang, 2018). As the recombinant protein was produced as a cleavable fusion to green fluorescent protein (sGFP) with a histidine tag, first-step isolation was achieved via native immobilized metal affinity chromatography (IMAC) (agarose resin derivatized with nickel ion-nitrilotriacetic acid; Qiagen, Germany). Following AcTEV^TM^ protease digestion (Thermo Fischer Scientific, United States), the final product (cEDIII alone) was harvested from the flow-through fraction of a second IMAC procedure. The cEDIII protein was dialysed against phosphate-buffered saline (PBS) prior to the downstream testing.

### Western Blotting Analysis

For immunoblotting analysis, the electrophoresed proteins were electroblotted onto a nitrocellulose membrane (GE Healthcare, United States) via wet transfer at 100 V for 1 h. Blotted membrane was blocked with 5% (w/v) milk powder in PBS containing 0.05% (v/v) Tween-20 (PBST) for at least 1 h. The membrane was subsequently incubated with either mouse anti-HBcAg monoclonal antibody (10E11; Abcam, United Kingdom) (1:4,000 dilution) or mouse anti-DENV 1–4 monoclonal antibody [D1-11(3); Thermo Fisher Scientific, United States] (1:2,000 dilution) for 1 h. Then, the membrane was washed three times with PBST at 5-min intervals before incubation with mouse immunoglobulin G (IgG) horseradish peroxidase (HRP)-conjugated secondary antibody (M30107; Invitrogen, United States) (1:10,000 dilution). Washing steps were repeated three times before the blot was soaked in chemiluminescence solution and detected using ImageQuant LAS 500 (GE Healthcare, United States).

### Transmission Electron Microscopy (TEM) Examination

All VLP samples (tHBcAg-cEDIII) were dialyzed against PBS using Float-A-Lyzer (Sigma, United States). Approximately 10 μl of the VLPs sample was adsorbed onto copper-palladium grids, washed with sterile distilled water and negatively stained with 2% (w/v) uranyl acetate. As an experimental control, the tandem core particles without gene insert (tEL, Peyret et al., 2015) was also examined. Both particles (tHBcAg-cEDIII and tEL) were viewed using the FEI Tecnai 20 transmission electron microscope (FEI, United States).

### Protein Quantification

Pierce modified Lowry protein assay kit was used according to the manufacturer’s instruction (Thermo Fisher Scientific, United States) for protein quantification. The absorbance of sample replicates and diluted albumin standards was then measured at 750 nm using CLARIOstar microplate reader (BMG LABTECH, Germany). The purified tHBcAg-cEDIII VLPs were also analyzed by SDS-PAGE (refer to Supplementary Figure S1 for this SDS-PAGE profile).

### Mouse Immunization

Two independent animal immunization experiments were conducted using female BALB/c mice (Envigo, United Kingdom) at 6–8 weeks old. For each experiment, at least four mice per group were immunized with 5 μg of purified VLP particles containing the cEDIII antigen insert (tHBcAg-cEDIII) mixed with 50 μl of Imject Alum (Thermo Fisher Scientific, United States) in a final volume of 100 μl made up by sterile saline solution. As a positive control group for cEDIII antigen, mice were immunized with 5 μg of recombinant cEDIII protein purified by using nickel ion-nitrilotriacetic acid agarose resin (Qiagen, Germany). Another group of mice was immunized with 5 μg of empty VLPs without cEDIII insert (tEL), which served as a negative control for cEDIII antigen. In addition, there was another control group of mice were injected with 100 μl of sterile normal saline solution. Three immunizations were delivered intra-peritoneally, administered 1 week apart. Tail bleeds were performed to check for seroconversion 4 weeks after primary immunization, and terminal bleeds at the completion of experiment at 9 weeks post-immunization. All animal works were done in compliance with United Kingdom Home Office approved animal protocols under license PPL 70/7376.

### Enzyme-Linked Immunosorbent Assay (ELISA)

Detection of antigen-specific IgG antibodies was performed by ELISA. Briefly, 96-well Nunc Maxisorp plates (Sigma, United States) were coated with 1 μg/ml pure cEDIII protein in carbonate bi-carbonate buffer overnight at 4°C. Coated plates were washed three times with PBS-Tween 20 (0.05% v/v) and blocked with 10% skimmed milk solution (Sigma, United States) for 1 h at 37°C. Primary sera were serially diluted (twofold) in 2.5% (w/v) milk solution from 1:100 to 3,200. All samples were added to duplicate test wells, incubated at 37°C for 1 h then washed three times as mentioned before. Goat-anti-mouse IgG-peroxidase secondary antibody was added at 1:2,500 dilution (Sigma, United States) and incubated at 37°C for 1 h then washed three times again. 3, 3′, 5, 5′-Tetramethylbenzidine substrate (Sigma, United States) was added to each well for 20 min and reaction was stopped with 1M H_2_SO_4_. Absorbance at 450 nm with a 630 nm correction was read using the SpectraMAX 190 plate reader (Molecular Devices, United States). Unpaired Student’s *t*-test was conducted to compare the immunization groups of mice receiving either recombinant cEDIII protein or tHBcAg-cEDIII VLPs against the empty tandem core VLPs control (tEL) in order to determine the significance level of cEDIII-specific IgG produced. The significant difference of cEDIII-specific IgG levels between cEDIII and tHBcAg-cEDIII groups was also compared. The significance levels were denoted at *p* ≤ 0.001 with ^∗∗^ and *p* ≤ 0.0001 with ^∗∗∗^ in the graph.

## Results

### Expression of Recombinant tHBcAg-cEDIII Construct in *N. benthamiana*

**FIGURE 2:**
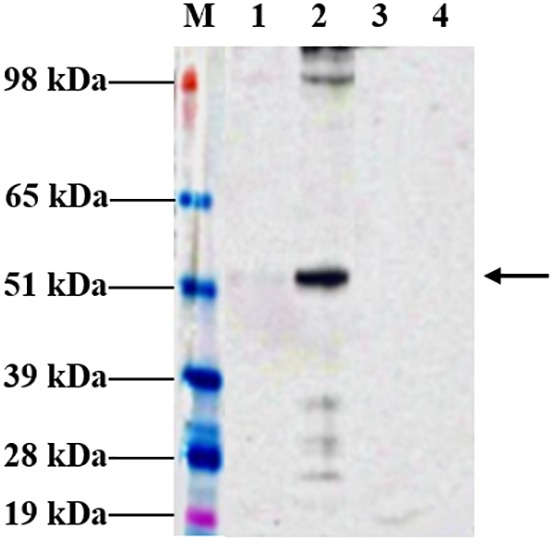
Expression profiles of tHBcAg-cEDIII protein in *N. benthamiana* analyzed at 6 dpi, which was predominantly obtained in insoluble form. Immunoblot detection of tHBcAg-cEDIII proteins at ∼54 kDa in size (black arrow) was performed using anti-HBcAg monoclonal antibody. Lane M: SeeBlue^®^ Plus2 Pre-Stained Standard; Lane 1: SP extracted from pEAQ-*HT*::tHBcAg-cEDIII-infiltrated leaf disks; Lane 2: IP extracted from pEAQ-*HT*::tHBcAg-cEDIII-infiltrated leaf disks; Lane 3: SP extracted from pEAQ-*HT*-infiltrated leaf disks; Lane 4: IP extracted from pEAQ-*HT*-infiltrated leaf disks.

Following agroinfiltration, *N. benthamiana* leaves were collected on 6 dpi and analyzed by Western blotting technique to check for expression. Western profiles indicated that the tHBcAg-cEDIII proteins had been expressed well in *N. benthamiana* infiltrated with pEAQ-*HT*::tHBcAg-cEDIII (Figure 2), based on the band at around 54 kDa which was not present in the pEAQ-*HT*-infiltrated plant leaf sample. However, the yield of soluble tHBcAg-cEDIII (SP) was very low (barely detectable) compared to the insoluble tHBcAg-cEDIII (IP) fraction. It was estimated that over 90% of the tHBcAg-cEDIII produced by the plant was insoluble.

### Kinetic Expression and Physical Observations on the Infiltrated *N. benthamiana*

**FIGURE 3:**
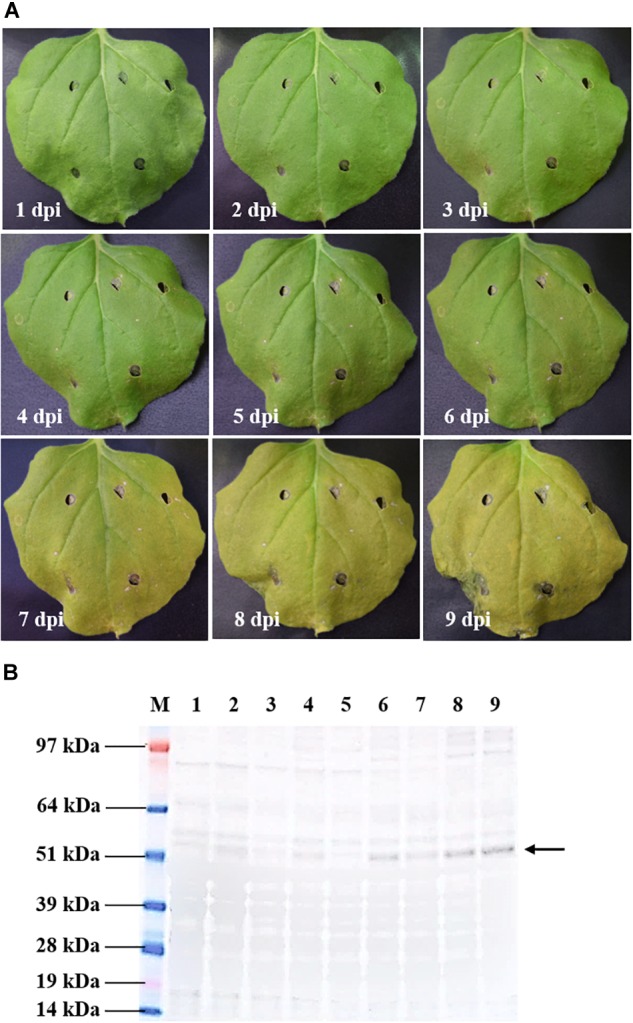
Kinetic study of pEAQ-*HT*::tHBcAg-cEDIII-infiltrated *N. benthamiana*. **(A)** Physical appearances of the representative infiltrated leaf from 1 to 9 dpi. **(B)** Expression profiles of the corresponding tHBcAg-cEDIII proteins (∼54 kDa; black arrow) which were immunoblotted with anti-HBcAg monoclonal antibody. Lane M: SeeBlue^®^ Plus2 Pre-Stained Standard; Lanes 1–9: SP extracted from infiltrated *N. benthamiana* from 1 to 9 dpi, correspondingly. It was decided that 8 dpi to be the suitable harvest time for soluble tHBcAg-cEDIII protein.

A time-course evaluation was performed to determine the optimal harvest time of tHBcAg-cEDIII protein for subsequent extraction and purification procedures. In general, symptoms of leaf chlorosis were observed from 7 dpi onward in all pEAQ-*HT*::tHBcAg-cEDIII-infiltrated plants, which led to visible necrosis by 9 dpi. This leaf chlorosis symptom was not observed with the empty vector, pEAQ-*HT*-infiltrated plants. A representative series of a pEAQ-*HT*::tHBcAg-cEDIII-infiltrated leaf is shown in Figure 3A. Concurrently, infiltrated leaves were harvested on a daily basis to monitor the accumulation of soluble proteins. As illustrated in Figure 3B, increasing amounts of tHBcAg-cEDIII SP could be seen from 6 dpi onward. As advanced necrosis was observed on 9 dpi in these infiltrated plants, it was deemed sensible to set 8 dpi as the optimal harvest time for soluble tHBcAg-cEDIII protein.

### Purification of Chimeric Tandem Core Particles Displaying cEDIII Epitopes

**FIGURE 4:**
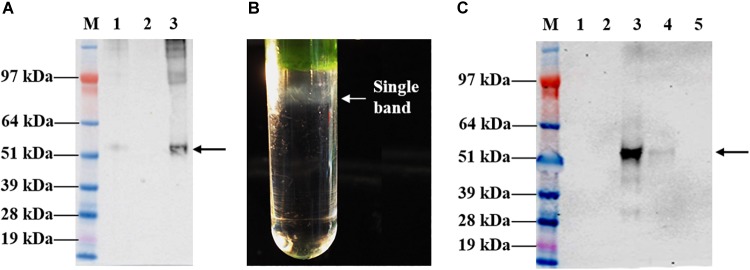
Sucrose cushion and Nycodenz gradient fractionation of proteins extracted from pEAQ-*HT*::tHBcAg-cEDIII-infiltrated leaves. **(A)** Western blot profiles showing the desired tHBcAg-cEDIII VLPs (black arrow) were successfully detected using anti-HBcAg monoclonal antibody. Lane M: SeeBlue^®^ Plus2 Pre-Stained Standard; Lane 1: Clarified plant lysate; Lane 2: 70% sucrose fraction; Lane 3: Interface fraction. **(B)** A single grayish band along the Nycodenz gradient that can be visualized. **(C)** Western blot profiles of Nycodenz gradient fractions detected using anti-DENV 1–4 monoclonal antibody. Lane 1: 60% Nycodenz fraction; Lane 2: 50% Nycodenz fraction; Lane 3: 40% Nycodenz fraction; Lane 4: 30% Nycodenz fraction; Lane 5: 20% Nycodenz fraction. As indicated by the black arrow, desired tHBcAg-cEDIII VLPs were successfully recovered from the infiltrated *N. benthamiana*.

Leaves infiltrated with the recombinant vector, pEAQ-*HT*::tHBcAg-cEDIII were harvested on 8 dpi for large-scale purification. The first isolation step via sucrose cushion yielded 70% sucrose and interface fractions, which were collected for maximal recovery of VLPs present in the sample (Figure 4A). Further purification by Nycodenz gradient gave rise to a single grayish band (Figure 4B). From here, the band was separated from the sedimentation of green impurities toward the top of the gradient. The single band was confirmed to be tHBcAg-cEDIII VLPs distribution following Western blotting analysis (Figure 4C), with sedimentation point estimated at around 40% Nycodenz concentration. The yield of purified tHBcAg-cEDIII VLPs was in the range of ∼12–16 mg/kg, which is comparable to that of purified recombinant cEDIII (∼13–14 mg/kg).

**FIGURE 5:**
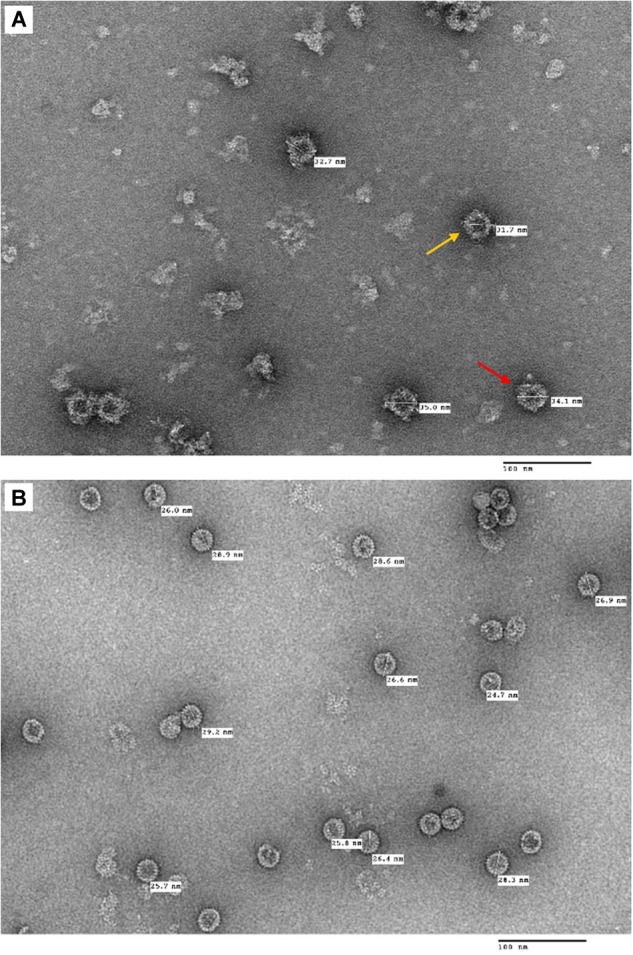
Electron micrographs showing the purified tandem core tHBcAg-cEDIII and tEL VLPs that were negatively stained with 2% (v/v) uranyl acetate. **(A)** tHBcAg-cEDIII VLPs visualized at 50,000× magnification. **(B)** Negative control tEL VLPs visualized at 50,000× magnification. Scale bar represents 100 nm. The chimeric tHBcAg VLPs with cEDIII epitopes had successfully assembled into viral particles.

Following the purification process, TEM imaging analysis revealed that plant-produced tHBcAg-cEDIII assembled into VLPs (Figure 5A), which were visualized as a mixture of core-like particles of slightly different sizes. These particles exhibited an irregular spherical morphology that characterizes tandem core particles displaying a heterologous sequence in the c/e1 loop. The diameters of these particles ranged from 32 to 35 nm, with an average particle size of ∼34 nm. As illustrated in Figure 5B, purified tEL sample (empty tandem core VLPs without insert) formed smaller, more evenly sized particles, with an average size of ∼27 nm in diameter. Comparatively, tHBcAg-cEDIII particles appeared to be somewhat larger than the empty tEL particles.

### Immunogenicity of Chimeric Tandem Core Particles Displaying cEDIII Epitopes

**FIGURE 6:**
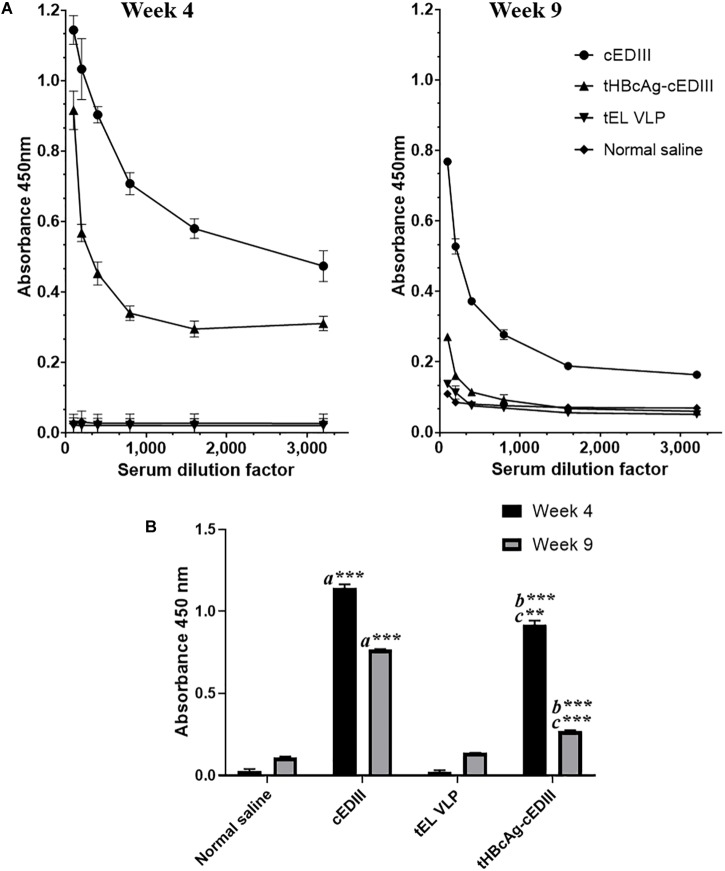
Evaluation of cEDIII-specific IgG antibody responses in BALB/c mice by using ELISA. **(A)** cEDIII-specific IgG antibody levels of mouse sera at the dilutions from 1:100 to 1:3,200 following 4- and 9-week post-immunizations. **(B)** Comparison of mouse antibody responses following 4- and 9-week post-immunizations as tested by ELISA at the dilution of 1:100. Bars represent the mean ± standard error of mean and the graphs are representative of two independent animal immunization experiments (*n* = 2). Student’s *t*-test was used to determine the significance levels (*p* ≤ 0.001 denotes as ^∗∗^ and *p* ≤ 0.0001 denotes as ^∗∗∗^). Note that *a* indicates the comparison between the immunization groups of mice receiving cEDIII (positive control) against empty VLPs (tEL; negative control); *b* indicates the comparison between tHBcAg-cEDIII and tEL VLPs and *c* indicates the comparison between cEDIII and tHBcAg-cEDIII groups.

The seroconversion results in BALB/c mice immunized with the VLP-based dengue vaccine candidate are presented in Figure 6. cEDIII-specific IgG antibody was successfully detected in mice immunized with tHBcAg-cEDIII VLPs and recombinant cEDIII protein alone. However, the IgG antibody level elicited by the recombinant cEDIII protein alone was higher than that of tHBcAg-cEDIII VLPs at all 1:100 to 1:3,200 dilutions tested (Figure 6A). The control groups (normal saline and tEL) did not trigger any cEDIII-specific IgG antibody responses and this observation has indicated the absence of pre-exposure of DENV (cEDIII). Figure 6B shows that cEDIII-specific IgG antibody level elicited by tHBcAg-cEDIII VLPs was significantly higher than that of the negative control, tEL-immunized mice (*p* ≤ 0.0001 at week 4 and week 9). However, when comparing between cEDIII and tHBcAg-cEDIII groups, the specific IgG level of cEDIII-immunized group mice was in fact significantly higher (*p* ≤ 0.001 at week 4 and *p* ≤ 0.0001 at week 9). In general, our data showed that IgG antibody levels appeared to peak after 4-week post-immunization, then began to wane but were still detectable more than 2 months after immunization. These results suggest that the cEDIII antigen inserted into the c/e1 loop of tandem core particles was still immunogenic and able to elicit a specific humoral response.

## Discussion

Development of VLPs as a highly structured form of subunit vaccine has been increasingly explored in recent years, whereby they present themselves as multimeric structures that mimic native virions. Production of dengue VLPs had been attempted previously (Sugrue et al., 1997; Liu et al., 2010; Zhang et al., 2011; Mani et al., 2013); however, several shortfalls were identified such as the neutralizing antibody responses acquired by mice were generally weak and co-expressing the DENV prM and EDI/EDII can trigger antibody-dependent enhancement (ADE) phenomenon. This is where pre-existing, non-neutralizing antibodies from an initial DENV infection can bind with the new infecting serotype and infect Fc gamma receptor (FcγR)-bearing cells to gain entry into host cells (Bäck and Lundkvist, 2013). Hence, ADE is often affiliated with disease aggravation and known to be the strongest risk factor of DHF/DSS development (Kliks et al., 1989). Therefore, generating a durable immunity against all four serotypes of DENV by using serotype-specific VLPs would require the expression of four monovalent VLPs together in the optimal tetravalent formulation, which would represent a significant technical challenge.

Because of this, the rationale for the work presented here was to produce chimeric VLPs which are able to self-assemble while presenting a consensus DENV antigen on their surfaces. In this study, the VLPs derived from HBcAg were utilized for cEDIII epitope display. As the stand-alone stability of DENV domain III has made it intrinsically different from other parts of the E protein (Soares and Caliri, 2013), it is believed that cEDIII can behave as an independent entity and would not interrupt the assembly of viral particles (Pang, 2018). The resulting chimeric HBcAg VLPs are therefore expected to display a high density of cEDIII epitopes on 90 or 120 copies of core protein dimers per particle.

In this study, “Tandem Core” technology was adopted due to the concern that two copies of cEDIII inserts at HBcAg dimer interface might suffer from steric clashes which could abrogate particle assembly. Thus, it is anticipated that this strategy could resolve the steric constraints as VLPs are now assembled from the dimers of HBcAg protein, expressed from a single open reading frame coding for two copies of HBcAg that have been covalently linked (Peyret et al., 2015). The cEDIII gene was inserted into the immunodominant c/e1 loop of Core II (the C-terminal copy of HBcAg), so that this would minimize the disruption to VLPs assembly as the translation moved in 5′ → 3′ direction from the unmodified Core I. With this, the tandem core would have greater flexibility as only one of the two c/e1 loops on each dimer was decorated with cEDIII antigens (Pang, 2018).

The *cEDIII* gene expressed in this study was codon-optimized based on *N. benthamiana* preference to boost translation by re-coding the rare codon in foreign gene with synonymous codon preferred by the expression host (Angov, 2011). Apart from that, a glycine and serine-rich linker was designed to avoid spurious interaction between the cEDIII inserts and core protein subdomains: this linker consisted of 15 amino acids on either side of the cEDIII insert (the exact sequence is shown in the Supplementary Material). As this approach was shown to be useful by Kratz et al. (1999), it was hoped that the (GGS)n linkers can help to minimize the steric constraints and stabilize the chimeric HBcAg VLPs expressing the cEDIII epitopes (Pang, 2018). Indeed, these linkers allowed the display of a nanobody protein inserted in Core II of plant-produced tandem cores (Peyret et al., 2015). Nevertheless, the length of the linkers was not optimized in the context of this work, and it is possible that higher yield and better solubility could be achieved upon optimization.

Based on the kinetic expression studies (Figure 3), it is postulated that the chimeric VLP construct does not confer significant toxicity to plants as early necrosis or apoptosis was not evident. The profiles were useful to gauge the ideal harvest time which is assessed based on the peak accumulation of soluble target protein, and the post-infiltration morphological distortions of the leaves. Necrotic tissues are usually avoided as they are flaccid and may contain higher amount of phenolics that could be introduced into downstream processing (Tanguy and Martin, 1972). In fact, the antimicrobial exudate produced by necrotic tissues can inhibit efficient colonization and gene delivery by *Agrobacterium* (Pitzschke, 2013). In this case, the optimal harvest time for the chimeric tHBcAg-cEDIII VLPs was determined to be around 8 dpi. A constant OD_600_ of 0.4 for the agrobacterial infiltration suspension was used throughout this study in line with the recommended range of OD_600_ at 0.3–0.4 as reported previously (Li, 2011; Pua et al., 2012; Shamloul et al., 2014). This is because high bacterial density tends to trigger hypersensitive response that can lead to tissue necrosis, whereas low amount of *Agrobacterium* may result in insufficient gene delivery (Leuzinger et al., 2013). As presented in this study, an acceptable level of transient gene expression was achieved at the chosen OD_600_ of 0.4, without severely triggering the hypersensitivity responses (Pang, 2018).

To purify the VLPs of interest, the procedures begin with a discontinuous two-step sucrose cushion to enrich the isolation of core particles from clarified lysate in a fast and reproducible manner (Peyret, 2015). After that, an additional isopycnic gradient was applied to complement the earlier technique in preparation of high purity particles (Brakke, 1961). Generally, Nycodenz is an inert chemical that can generate a self-forming gradient (Gugerli, 1984) and it worked well for the purification of tHBcAg-cEDIII VLPs as evidenced in Figure 4. TEM observation revealed that the assembly of particles was successful (Figure 5A). Instead of being uniformly shaped, tHBcAg-cEDIII particles appeared to be rather “knobbly” due to the epitopes protruding from HBcAg spikes. These VLPs, which ranged from 32 to 35 nm in diameter are somewhat larger than their empty counterpart labeled as tEL (Figure 5B). This type of surface morphology is in fact expected for tandem core particles displaying a heterologous sequence on their surface (Peyret et al., 2015). Such finding indicates that cEDIII epitopes were presented on the protruding spikes of HBcAg that retained the inherent propensity to fold into discrete VLPs. In fact, the size range of 35–40 nm for chimeric HBcAg VLPs with dengue EDIII epitopes produced in microbial cells, was previously reported by Arora et al. (2012, 2013).

To our knowledge, this is the first study that reports the successful production of chimeric HBcAg VLPs with dengue protein epitopes in a plant system. It was shown that the cEDIII epitopes remain immunogenic when presented on the VLP scaffold (HBcAg-cEDIII): the specificity of the IgG responses detected is demonstrated by the lack of cEDIII-reactive antibodies in the saline control and empty tHBcAg (tEL) groups (Figure 6). The specific IgG antibody levels induced by both HBcAg-cEDIII VLPs and recombinant cEDIII (positive control) were higher at week 4 post-immunization and declined thereafter as detected at week 9, which is consistent with the kinetics of a normal immune response (Leo et al., 2011). As the ELISA plates were coated with purified cEDIII protein, it is possible that the higher IgG antibody level detected in the recombinant cEDIII group could be due to an optimum antigen-antibody match as compared to that of tHBcAg-cEDIII group. Additionally, the low antibody response (tHBcAg-cEDIII) could also be explained by the lower molar antigen dose received by the HBcAg-cEDIII-immunized mice as the antigen dose for immunization was normalized to a total protein concentration of 5 μg. Therefore, the actual dosage of cEDIII on the VLPs is much lower (in molar terms) than the subunit recombinant proteins. The tHBcAg-cEDIII is composed mainly of tHBcAg carrier by protein mass, and cEDIII only contributes to 103 out of the total 508 amino acids. Taken the suggestion of Whitacre et al. (2016), the amount of cEDIII presented in relation to the entire chimeric particle size should be considered in future in order to gauge the optimal dose needed for *in vivo* study.

In any case, the results presented here highlight potential limitations to chimeric VLP vaccine development strategies. The recovered yield of tHBcAg-cEDIII VLPs is considered relatively low, as it was estimated that less than 10% of the tHBcAg-cEDIII protein formed soluble particles. Extraction under denaturing conditions was not attempted, since denaturing and refolding steps for such a complex structure (an assembly of 90 or 120 copies of a triple protein fusion) are unlikely to result in the proper formation of core-like particles. In this study, the tHBcAg-cEDIII protein was targeted to the cytosol, since this is the strategy that had previously been used successfully with tandem cores (Peyret et al., 2015), whereas, by contrast, the recombinant cEDIII protein alone was targeted to the endoplasmic reticulum (ER). Subcellular localization for tHBcAg-cEDIII protein was not attempted initially due to the concerns that distinct pH conditions in different plant organelles could abolish VLPs assembly as shown by van Zyl et al. (2016). Nevertheless, it has recently been shown that HBcAg VLPs are still capable of assembly despite localization to ER (Yang et al., 2017). In fact, accumulation of tHBcAg-cEDIII VLPs in the plant cytosol may have affected the antigen stability via improper disulphide bond formation in cEDIII. This issue is highlighted because the correct folding of antibody recognition epitope on DENV EDIII relies upon the disulphide linkage (Suzarte et al., 2014). Given that the redox environment is highly regulated in the ER (Ellgaard, 2004), optimal oxidoreductase activity can be achieved to form proper disulphide bridges. The relatively low antibody response generated in mice immunized with tHBcAg-cEDIII VLPs as compared to recombinant cEDIII immunized group might therefore also be explained by the presence of VLPs with improper folding produced in the plant cytosol.

Hence, as the way forward, future optimizations to modulate protein yield and stability at the post-translational level may include attempting different extraction buffers to improve the solubility of crude extract as well as targeting the heterologous protein to a subcellular compartment. Researchers carrying out the future works would be well-advised to test the pre-immune sera from each individual mouse in order to increase confidence in any post-immunization results. The full benefits of VLP presentation should be explored further, as its three-dimensional structure has a higher potency to activate cell-mediated immunity including the cytotoxic T cell response, which is crucial for better viral clearance as reported by Yang et al. (2017). Besides, since cEDIII represents a consensus antigen, it would also be of great interest to test the cross-reactivity of polyclonal responses as well as the protection levels of tHBcAg-cEDIII VLPs against the different DENV serotypes.

## Conclusion

While there has been a previous initiative that used a yeast system to produce a chimeric VLP-based dengue vaccine candidate (Arora et al., 2013); development of a plant-derived vaccine can offer scalability and safety advantages that revolutionize the accessibility of dengue vaccines (Pang and Loh, 2017). This is particularly important to address the alarming burden of dengue disease that has yet to meet a promising resolution. The adoption of “Tandem Core” technology was proven to be feasible although there is still room for improvements. Overall, a successful assembly of VLPs displaying a consensus dengue antigen has been achieved. The immunization data have shown that seroconversion to cEDIII is possible when mice are immunized with tHBcAg-cEDIII VLPs. The current findings have validated the viability of using a VLP system as an antigen-presentation platform; this warrants further investigation into its potential as a next-generation dengue vaccine.

## Ethics Statement

All animal works in this study were done in compliance with United Kingdom Home Office approved animal protocols under license PPL 70/7376.

## Author Contributions

H-SL, WMR, and GPL conceived and designed the study. ELP, HP, and AR performed the experiments. C-MF performed the statistical analysis. ELP and H-SL wrote the first draft of the manuscript. HP, AR, K-SL, and C-MF wrote sections of the manuscript. All authors contributed to manuscript revision and read and approved the current version.

## Conflict of Interest Statement

GPL declares that he is a named inventor on granted patent WO 29087391 A1 that describes the system used for transient expression in this manuscript. The Tandem Core vaccine technology described in this paper is covered by the patent application PCT/GB01/01607 licensed by iQur Limited. AR and WMR are the current employees of iQur Limited. The remaining authors declare that the research was conducted in the absence of any commercial or financial relationships that could be construed as a potential conflict of interest.
