# Supplementary material for: Epitope Presentation of Dengue Viral Envelope Glycoprotein Domain III on Hepatitis B Core Protein Virus-Like Particles Produced in Nicotiana benthamiana
Source: Front Plant Sci. 2019 Apr 16;10:455. doi: 10.3389/fpls.2019.00455 (PMC6477658; doi:10.3389/fpls.2019.00455)
Supplement: Supplementary file 1 [file Table_1.DOCX]

Epitope presentation of dengue viral envelope glycoprotein domain III on hepatitis B core protein virus-like particles produced in *Nicotiana benthamiana*

# Supplementary Materials

**(A) Sequence of tHBcAg-cEDIII Protein**

MDIDPYKEFGATVELLSFLPSDFFPSVRDLLDTASALYREALESPEHCSPHHTALRQAILCWGELMTLATWVGNNLEPGAGGSSGQLDPASRDLVVNYVNTNMGLKIRQLLWFHISCLTFGRETVLEYLVSFGVWIRTPPAYRPPNAPILSTLPETTVVGGSSGGSGGSGGSGGSGGSGGTGTMDIDPYKEFGATVELLSFLPSDFFPSVRDLLDTASALYREALESPEHCSPHHTALRQAILCWGELMTLATWVGNNLVDGGGGSGGGGSGGGGSPRKGMSYAMCTGKFKLEKEVAETQHGTILIKVKYEGDGAPCKIPFEIQDVEKKHVNGRLITANPIVTDKESPVNIEAEPPFGDSYIVIGVGDKALKLNWFKKGSSPAGGGGSGGGGSGGGGSINDPASRDLVVNYVNTNMGLKIRQLLWFHISCLTFGRETVLEYLVSFGVWIRTPPAYRPPNAPILSTLPETTVVRRRDRGRSPRRRTPSPRRRRSQSPRRRRSQSRESQC

**(B) Sequence of cEDIII Protein**

KGMSYAMCTGKFKLEKEVAETQHGTILIKVKYEGDGAPCKIPFEIQDVEKKHVNGRLITANPIVTDKESPVNIEAEPPFGDSYIVIGVGDKALKLNWFKKGSS


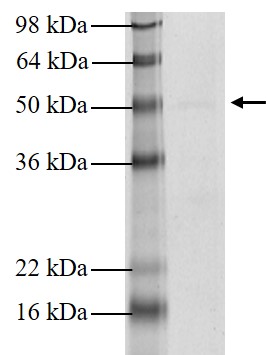


**Figure S1:** SDS-PAGE profile of the purified tHBcAg-cEDIII VLPs following protein quantification via modified Lowry assay. A faint but discernible band of the desired protein at ~54 kDa can be seen (black arrow).
